# Supplementary material for: Trifluoroacetate induced small-grained CsPbBr3 perovskite films result in efficient and stable light-emitting devices
Source: Nat Commun. 2019 Feb 8;10:665. doi: 10.1038/s41467-019-08425-5 (PMC6368619; doi:10.1038/s41467-019-08425-5)
Supplement: Supplementary file 1 — Supplementary Information [file 41467_2019_8425_MOESM1_ESM.pdf]

Supplementary Information

Trifluoroacetate induced small-grained $CsPbBr_3$ perovskite films result in efficient and stable light-emitting devices

Wang *et al.*

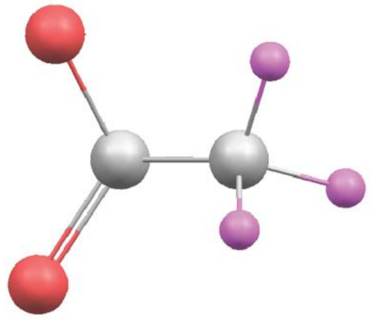

Supplementary Figure 1. Molecular structure of the TFA^- anion. Color coding: O (red), C (grey), and F (purple).

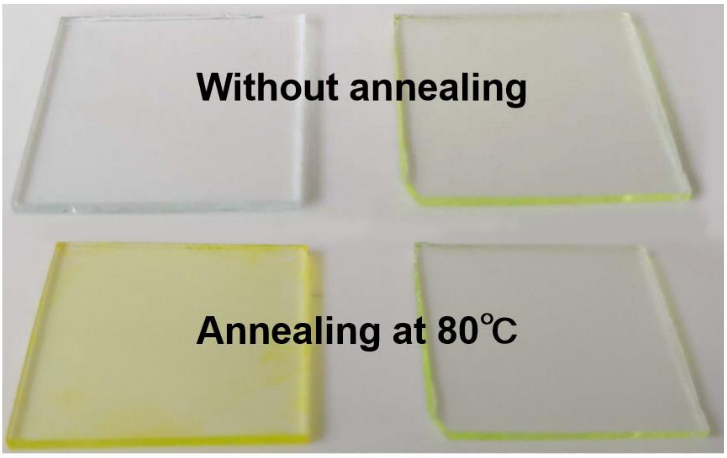

Supplementary Figure 2. Photographs of CsBr-derived (left column) and CsTFA-derived $CsPbBr_3$ (right column) films with and without thermal annealing at 80 ^oC.

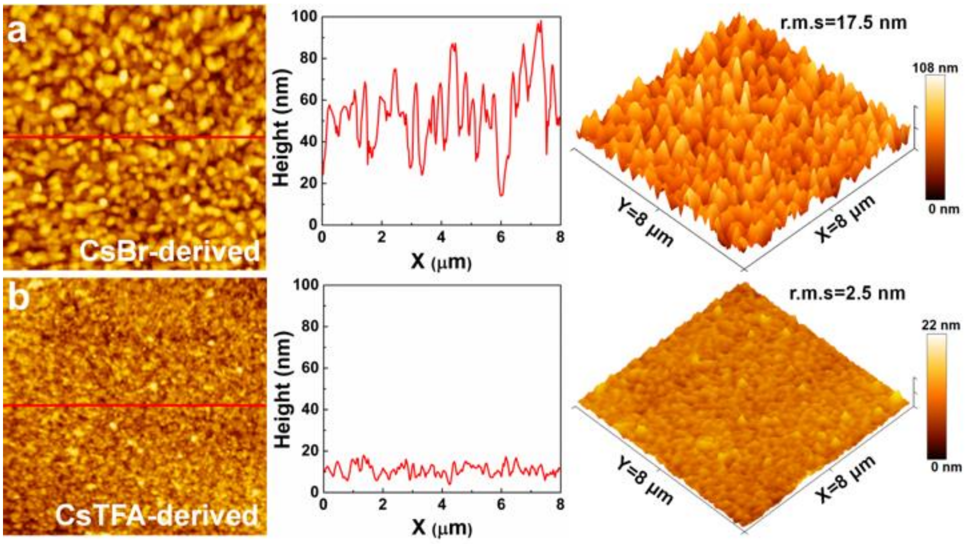

Supplementary Figure 3. AFM characterization of (a) ITO/PEDOT:PSS/CsBr(1.7)- and (b) ITO/PEDOT:PSS/ CsTFA(1.7)-derived perovskite films: height images (left), line-scan profiles (middle) and three-dimensional images (right).

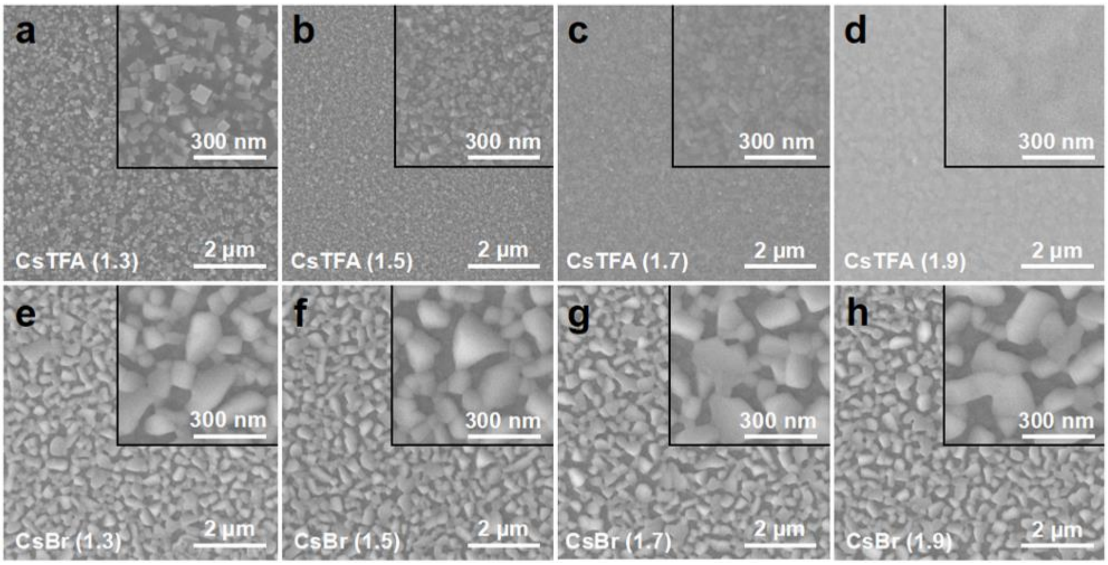

Supplementary Figure 4. FE-SEM images of perovskite films deposited on ITO/PEDOT:PSS substrates from the two different precursor solutions: (a-d) CsTFA, and (e-h) CsBr.

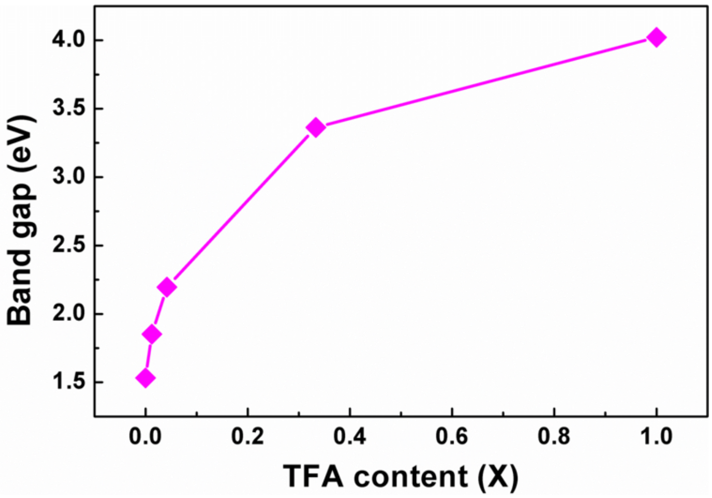

Supplementary Figure 5. Calculated band gaps of $CsPbBr_{3-x}(TFA)_x$ at different TFA concentrations.

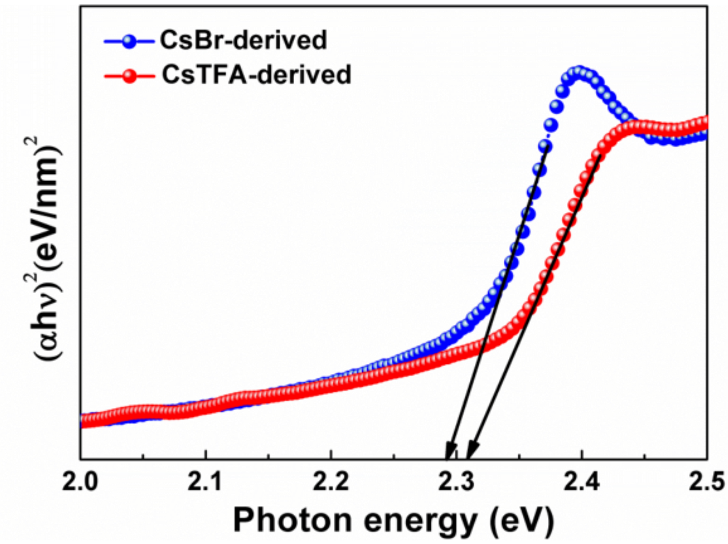

Supplementary Figure 6. Tauc plots showing the dependence of $(\alpha h\nu)^2$ of perovskite films upon the incident photon energy ($h\nu$) (assuming direct allowed transitions).

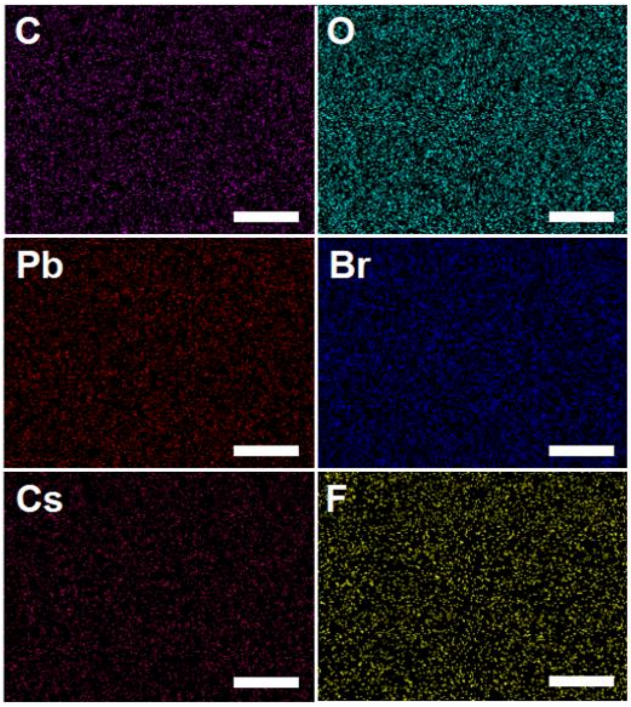

Supplementary Figure 7. EDX mapping showing homogeneous distribution of C, O, Pb, Br, Cs and F elements in CsTFA-derived $CsPbBr_3$ perovskite film. All scale bars are 2.5 μm.

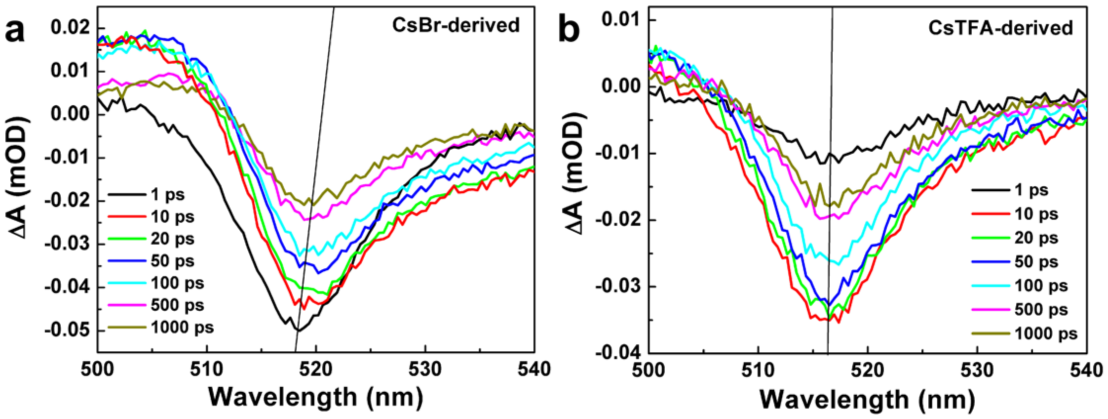

Supplementary Figure 8. Transient absorption spectra of (a) CsBr(1.7)- and (b) CsTFA(1.7)-derived films for different pump-probe delays.

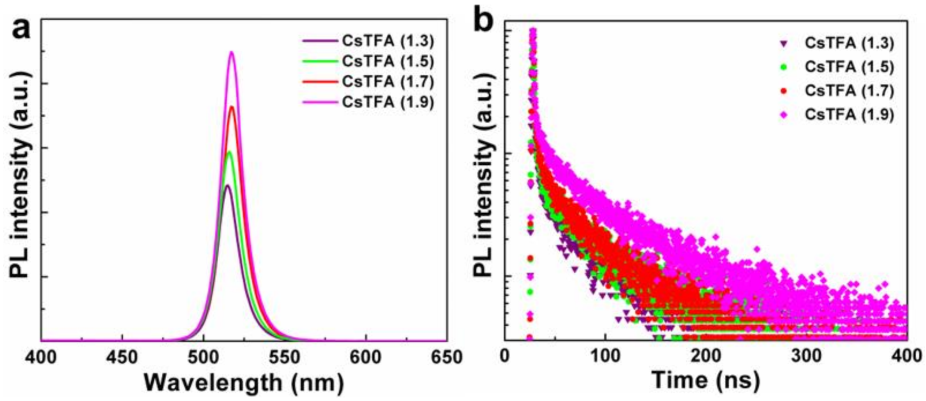

Supplementary Figure 9. (a) PL spectra, and (b) time-resolved PL decay curves of the perovskite films on ITO/PEDOT:PSS/substrates made from different molar ratios of $CsTFA:PbBr_2$.

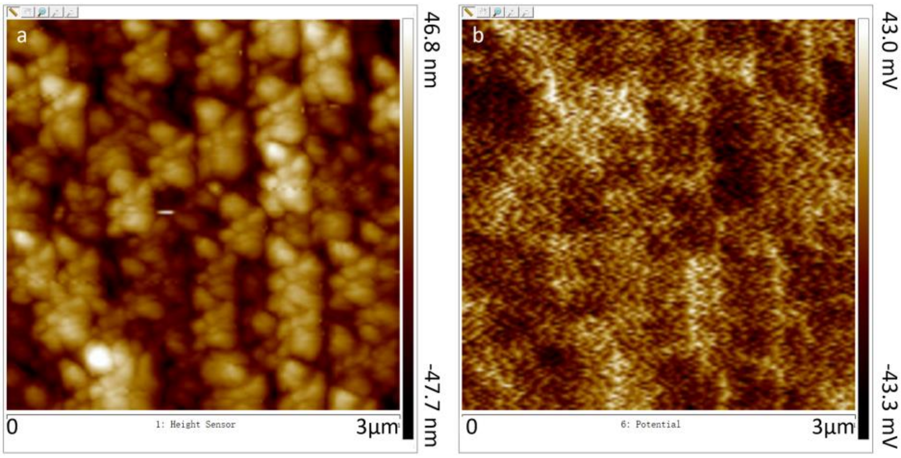

Supplementary Figure 10. (a) Topography map and (b) contact potential difference image of the TFA-derived $CsPbBr_3$ films.

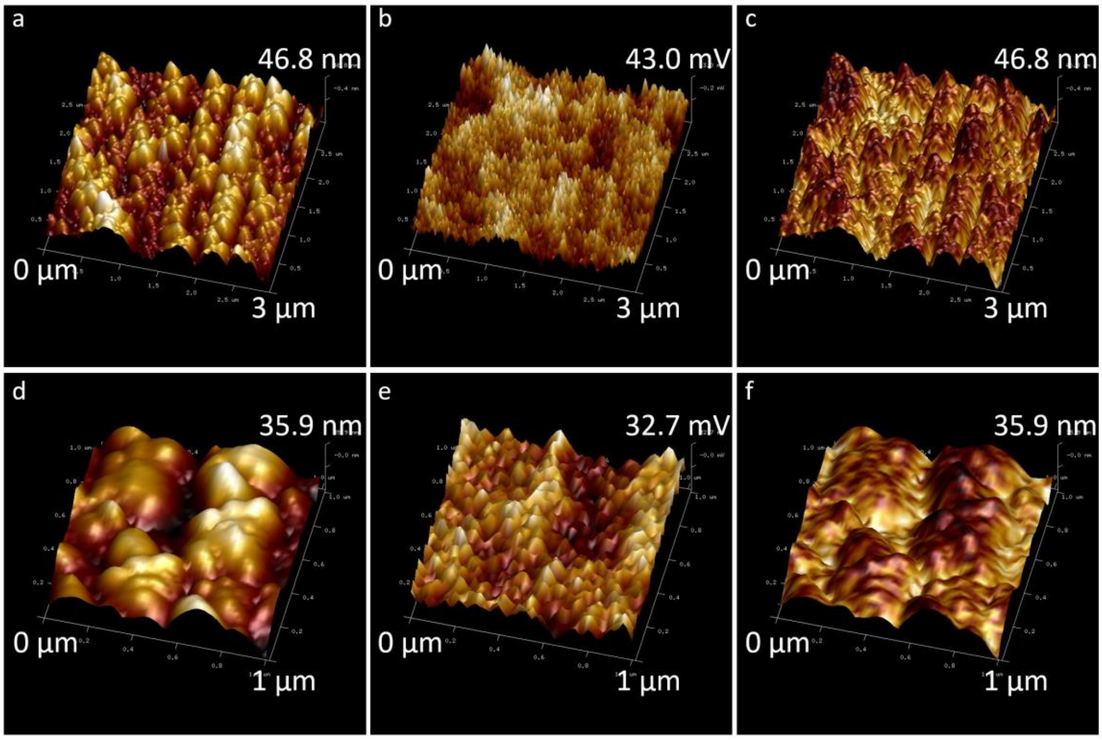

Supplementary Figure 11. (a,d) Topography, (b,e) contact potential difference, and (c,f), their overlap 3D images for differently sized TFA-derived $CsPbBr_3$ films.

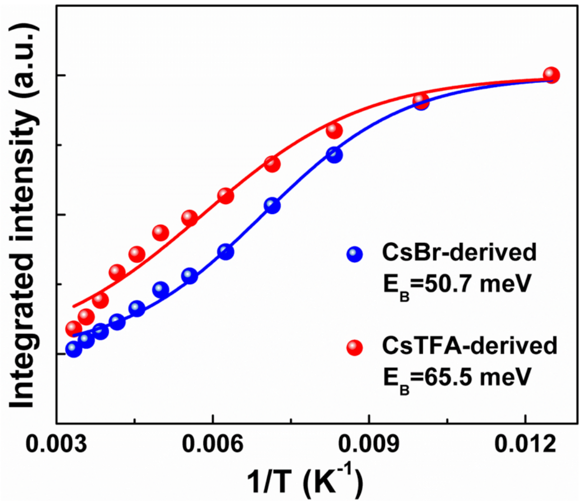

Supplementary Figure 12. Integrated PL emission intensity as a function of temperature from 80 to 300 K for CsTFA-derived and CsBr-derived films. The CsBr(1.7) and the CsTFA(1.7) samples were chosen for the temperature-dependent photoluminescence (PL) measurement to compare the exciton binding energy difference, as shown in **Supplementary Figure 12.** We determined the exciton binding energy (E_B) using the following equation:

$$I(T) = \frac{I_0}{1 + Ae^{(-E_B/k_B T)}} \quad (1)$$

where I_0 is the emission intensity at 0 K, A is a scaling factor, and k_B is the Boltzmann constant. The calculated E_B values are 65.5 meV for the CsTFA-derived film and 50.7 meV for the CsBr-derived film.

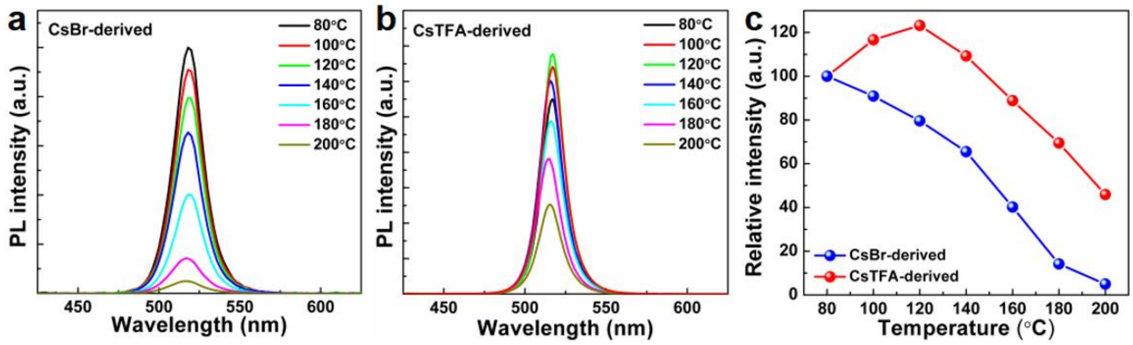

Supplementary Figure 13. Temperature-dependent PL evolution of (a) CsBr(1.7)- and (b) CsTFA(1.7)-derived perovskite films. Data points in Figure S11 (c) were determined according to the PL peak intensity of (a) CsBr(1.7)- and (b) CsTFA(1.7)-derived films at different temperature.

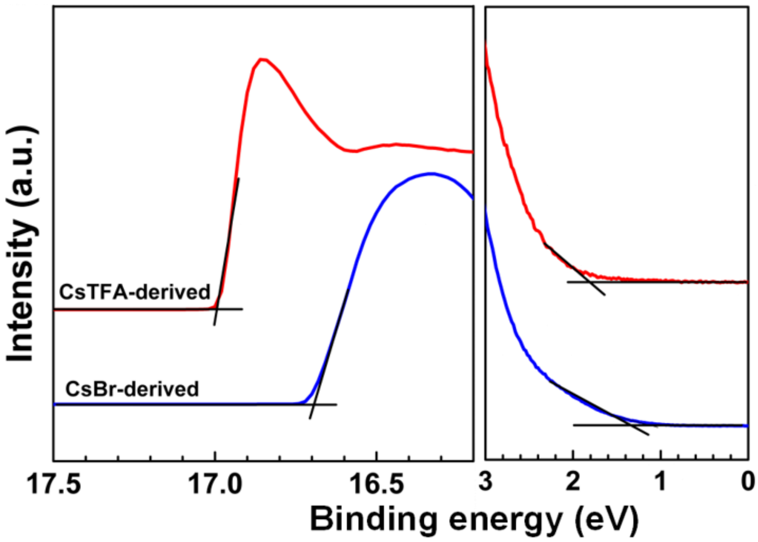

Supplementary Figure 14. UPS spectra of CsBr(1.7)- and (b)CsTFA(1.7)-derived perovskite films showing the binding energy secondary-electron cutoffs (left) and HOMO regions (right).

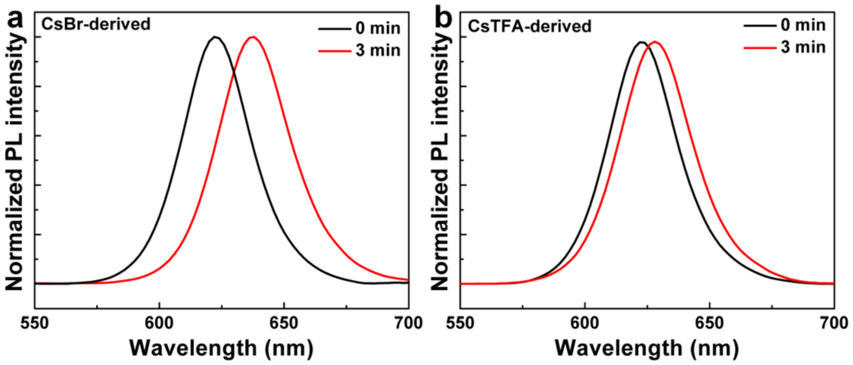

Supplementary Figure 15. Normalized PL spectra of the CsBr- and CsTFA-derived $CsPb(Br/I)_3$ devices under a constant bias of 2 V. PL spectra were recorded from 0 min to 3 min.

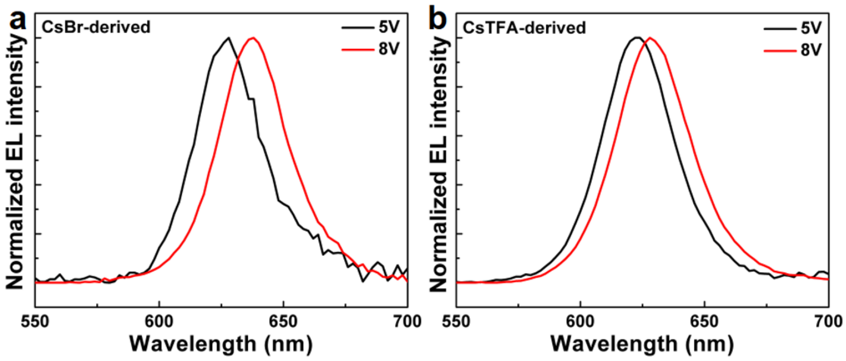

Supplementary Figure 16. Normalized EL spectra of (a) the CsBr- and (b) CsTFA-derived $CsPb(Br/I)_3$ devices. EL spectra were recorded from 5V to 8V. Since the bandgap changes over the halogen ratios for mixed-halide perovskites, we can determine the components based on the emission color. Thus, to provide a direct observation of ion migration, the mixed-halide $CsPb(Br/I)_3$ perovskites were taken, and their emission was traced in the LED structure of ITO/PEDOT:PSS/$CsPb(Br/I)_3$/TPBI/LiF/Al. First, the PL evolution over time under a constant voltage (2V, below the turn-on voltage) was investigated. As shown in **Supplementary Figure 15**, the PL spectra experience 15 and 6 nm red-shifts occurring within 3 min for CsBr- and CsTFA-derived $CsPb(Br/I)_3$ devices, respectively. The stronger PL shift of the CsBr-derived device indicates more intense ion migration within the film. In addition, the PL completely disappeared for the CsBr-derived device in 9 min, most probably due to destruction of perovskite structure or the formation of defects related to the halogen deficiency. In contrast to that, the CsTFA-derived $CsPb(Br/I)_3$ device was still shining brightly, because the ion migration was suppressed here. Besides, when the applied bias increased from 5 to 8 V, the EL peaks experienced 12 and 4 nm red-shifts for CsBr- and TFA-derived $CsPb(Br/I)_3$ perovskite LEDs, respectively (**Supplementary Figure 16**), which further demonstrates that the ion migration has been suppressed with the help of TFA ions.

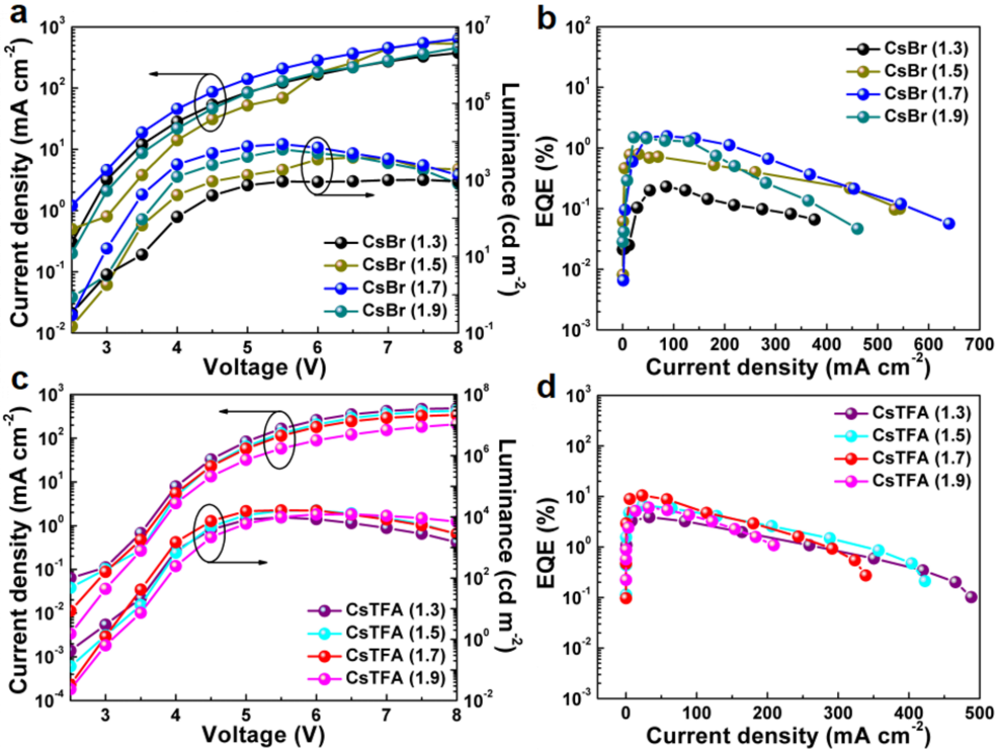

Supplementary Figure 17. (a) *J*-*V*-*L*, and (b) EQE-*J* curves of the PeLEDs based on perovskite films derived from different molar ratios of CsBr:$PbBr_2$. (c) *J*-*V*-*L*, and (d) EQE-*J* curves of the PeLEDs based on perovskite films derived from different molar ratios of CsTFA:$PbBr_2$.

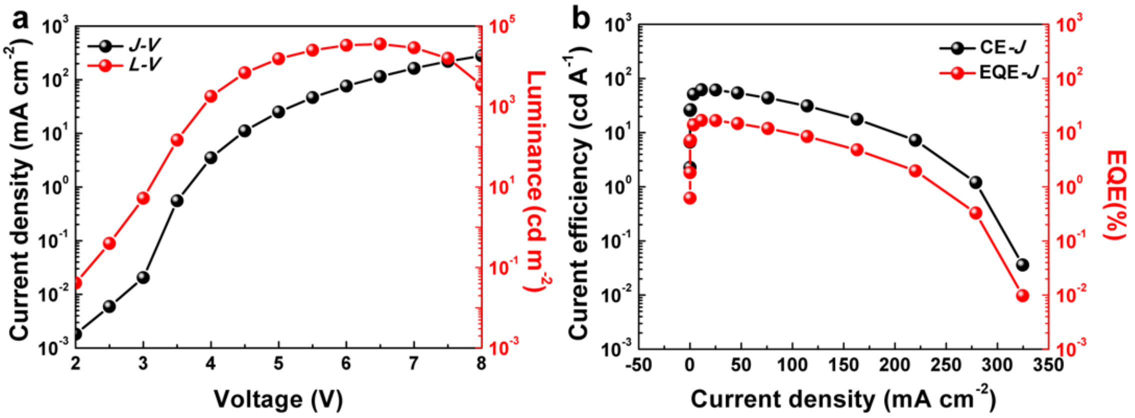

Supplementary Figure 18. (a) *J*-*V*-*L*, and (b) CE-*J*-EQE curves of the CsTFA-derived $FA_{0.11}MA_{0.10}Cs_{0.79}PbBr_3$ PeLED. TFA-derived mixed cation $FA_{0.11}MA_{0.10}Cs_{0.79}PbBr_3$ LEDs have shown a maximum luminance of 35,700 cd m^{-2}, and the peak EQE of 17%.

Supplementary Table 1. Calculated lattice constants of $CsPbBr_{3-x}(TFA)_x$.

	$CsPb(TFA)_3$	$CsPbBr_{3-x}$ $(TFA)_x$				$CsPbBr_3$	
		Super cell		Volume	A	A	Volume
a(Å)-100	6.804	1×1×1	6.114	228.61	6.1145	5.869	202.12
a(Å)-110	6.756	2×2×2	11.905	1687.41	11.9052	5.869*2 =11.738	
a(Å)-111	6.656	3×3×3	17.852	5690.14	17.8528	5.869*3 =17.607	

Supplementary Table 2. Calculated band gap of $CsPbBr_{3-x}(TFA)_x$.

$CsPb(TFA)_3$	$CsPbBr_{3-x}(TFA)_x$		$CsPbBr_3$
Gap (eV)	Super cell	Gap (eV)	Gap (eV)
4.022	1×1×1	3.363	1.532
-	2×2×2	2.196	-
-	3×3×3	1.852	-

Supplementary Table 3. PL lifetimes and PL QYs of the CsBr- and CsTFA-derived perovskite films.

Sample	τ_1 [ns]	A_1 [%]	τ_2 [ns]	A_2 [%]	τ_3 [ns]	A_3 [%]	τ_{avg} [ns]	QY [%]
CsBr(1.7)	1.21	68.10	7.86	6.62	50.67	25.28	46.31	19.2
CsTFA(1.3)	1.03	24.07	13.00	20.37	68.37	55.56	64.36	50.3
CsTFA(1.5)	0.88	22.20	11.07	16.43	71.55	61.38	68.84	53.6
CsTFA(1.7)	0.73	19.14	9.30	17.46	73.79	63.41	71.43	57.2
CsTFA(1.9)	0.97	11.09	18.06	21.12	93.32	67.79	88.90	60.2

Supplementary Table 4. Energy levels of CsBr(1.7)- and CsTFA(1.7)-derived perovskite films.

Samples	VBM (eV)	CBM (eV)
CsBr(1.7)	-5.87	-3.58
CsTFA(1.7)	-5.95	-3.64

Supplementary Table 5. Summary of the performance characteristics of PeLEDs based on CsBr-derived perovskite films for the different molar ratios of $CsBr:PbBr_2$

Samples	Max. luminance [cd m^{-2}]	Max. CE [cd A^{-1}]	Max. EQE [%]
CsBr(1.3)	989	0.85	0.23
CsBr(1.5)	3828	2.93	0.80
CsBr(1.7)	8628	5.78	1.57
CsBr(1.9)	6238	5.54	1.50

Supplementary Table 6. Summary of the performance characteristics of PeLEDs based on CsTFA-derived perovskite films for the different molar ratios of CsTFA:$PbBr_2$

Samples	Max. luminance [cd m^{-2}]	Max. CE [cd A^{-1}]	Max. EQE [%]
CsTFA(1.3)	9696	11.69	3.88
CsTFA(1.5)	16168	20.24	6.68
CsTFA(1.7)	16436	32.00	10.51
CsTFA(1.9)	12189	18.82	6.02

Supplementary Note 1.

For the preparation of TFA-derived perovskite films, $PbBr_2$ and CsTFA were firstly dissolved in DMSO, producing Pb^{2+}, Br^-, Cs^+, and TFA^- ions. Due to the ionic nature of the lead-halide perovskites[1], the precursors gradually precipitate and grow into crystals as the DMSO evaporated. The formation equation should be $Cs^+ + Pb^{2+} + 3Br^- \rightarrow CsPbBr_3$ within the grains, and $Cs^+ + Pb^{2+} + (3\text{-}x)Br^- + xTFA^- \rightarrow CsPb(Br/TFA)_3$ ($0 \leq x \leq 3$) at the grain boundaries.

Supplementary Note 2.

One can obtain large perovskite crystals *via* (i) solvent annealing to decrease the crystal growth rate[2], and (ii) doping to increase the crystal formation energy and thus to decrease the crystal growth rate[3]. This is because when decreasing the perovskite crystal growth rate, the nucleation process results in formation of fewer seed crystals, which would consume more precursors, resulting in larger sized crystals. In comparison, if one increases the crystal growth rate, the nucleation process becomes faster and forms more seed crystals, which would then consume less precursors, resulting in smaller sized crystals. This has also been reported while introducing an antisolvent to increase the crystal growth rate during the perovskite formation, which has also decreased the crystal size[4]. Based on these observations, the conclusion is that TFA can lead to fiercer reaction, and the smaller crystals are simultaneously obtained in this case which is beneficial to the increased amount of seed crystals.

Supplementary Note 3.

Density functional theory (DFT) calculations using the VASP code and band gap measurements of perovskite films made via the CsBr and CsTFA routes.

(1) *The calculated ion radius of TFA with different methods*

Method I (Fully substituted in primitive cell):

$$\frac{R_{Pb}+R_{TFA}}{R_{Pb}+R_{Br}}=\frac{a_{CsPb(TFA)_3}}{a_{CsPbBr_3}} \tag{1}$$

The notation 'a_x' means the lattice constant of x with cubic phase.

R_{Pb}=1.19 Å, R_{Br}=1.96 Å, R_{TFA}=6.656×(1.19+1.96)/5.869-1.19=2.38 Å,

Method II (Partially substituted in super cell):

R_{Pb}=1.19 Å, R_{Br}=1.96 Å,

Super cell 1 ×1 ×1:

$$\frac{2R_{Pb}+2R_{TFA}}{2R_{Pb}+2R_{Br}}=\frac{a_{CsPbBr_{3-x}(TFA)_x}}{a_{CsPbBr_3}} \tag{2}$$

R_{TFA}=1.0418 ×(1.19+1.96)-1.19=2.09 Å

Super cell: 2 ×2 ×2:

$$\frac{4R_{Pb}+2R_{Br}+2R_{TFA}}{4R_{Pb}+4R_{Br}}=\frac{a_{CsPbBr_{3-x}(TFA)_x}}{a_{CsPbBr_3}} \tag{3}$$

R_{TFA}=0.5 ×[1.0142 ×(1.19+1.96) ×4-4 ×1.19-2 ×1.96] =2.05 Å

Super cell: 3 ×3 ×3:

$$\frac{6R_{Pb}+4R_{Br}+2R_{TFA}}{6R_{Pb}+6R_{Br}}=\frac{a_{CsPbBr_{3-x}(TFA)_x}}{a_{CsPbBr_3}} \tag{4}$$

R_{TFA}=0.5 ×[1.0139 ×(1.19+1.96) ×6-6 ×1.19-4 ×1.96]=2.09 Å

Supplementary Note 4.

All TRPL decay curves were well fitted with triexponential decay model as follows:

$$I=A_1 e^{-t/\tau_1}+A_2 e^{-t/\tau_2}+A_3 e^{-t/\tau_3}+I_0 \tag{5}$$

where I is the normalized PL intensity; A_i (i = 1, 2 and 3) and τ_i (i = 1, 2 and 3) are the fractions and lifetimes of the three decay components, respectively. I_0 is a baseline

constant. The average lifetime (τ_{avg}) can be calculated according to the following formula:

$$\tau_{\mathrm{avg}} = \frac{\sum A_i \tau_i^2}{\sum A_i \tau_i}, \ i\text{=1, 2 and 3} \quad (6)$$

Supplementary References

1. Li, X. et al. $CsPbX_3$ quantum dots for lighting and displays: room-temperature synthesis, photoluminescence superiorities, underlying origins and white light-emitting diodes. *Adv. Funct. Mater.* **26**, 2435-2445 (2016).
2. Xiao, Z. et al. Solvent annealing of perovskite-induced crystal growth for photovoltaic-device efficiency enhancement. *Adv. Mater.* **26**, 6503-6509 (2014).
3. Chiang, Y.-H., Li, M.-H., Cheng, H.-M., Shen, P.-S & Chen, P. Mixed cation thiocyanate-based pseudohalide perovskite solar cells with high efficiency and stability. *ACS Appl. Mater. Inter.* **9**, 2403-2409 (2017).
4. Cho, H. et al. Overcoming the electroluminescence efficiency limitations of perovskite light-emitting diodes. *Science* **350**, 1222-1225 (2015).
